# Histone modifications are responsible for decreased Fas expression and apoptosis resistance in fibrotic lung fibroblasts

**DOI:** 10.1038/cddis.2013.146

**Published:** 2013-05-02

**Authors:** S K Huang, A M Scruggs, J Donaghy, J C Horowitz, Z Zaslona, S Przybranowski, E S White, M Peters-Golden

**Affiliations:** 1 Division of Pulmonary and Critical Care Medicine, University of Michigan Medical School, Ann Arbor, MI, USA

**Keywords:** epigenetics, histone deacetylation, histone methylation, histone deacetylase

## Abstract

Although the recruitment of fibroblasts to areas of injury is critical for wound healing, their subsequent apoptosis is necessary in order to prevent excessive scarring. Fibroproliferative diseases, such as pulmonary fibrosis, are often characterized by fibroblast resistance to apoptosis, but the mechanism(s) for this resistance remains elusive. Here, we employed a murine model of pulmonary fibrosis and cells from patients with idiopathic pulmonary fibrosis (IPF) to explore epigenetic mechanisms that may be responsible for the decreased expression of Fas, a cell surface death receptor whose expression has been observed to be decreased in pulmonary fibrosis. Murine pulmonary fibrosis was elicited by intratracheal injection of bleomycin. Fibroblasts cultured from bleomycin-treated mice exhibited decreased Fas expression and resistance to Fas-mediated apoptosis compared with cells from saline-treated control mice. Although there were no differences in DNA methylation, the Fas promoter in fibroblasts from bleomycin-treated mice exhibited decreased histone acetylation and increased histone 3 lysine 9 trimethylation (H3K9Me3). This was associated with increased histone deacetylase (HDAC)-2 and HDAC4 expression. Treatment with HDAC inhibitors increased Fas expression and restored susceptibility to Fas-mediated apoptosis. Fibroblasts from patients with IPF likewise exhibited decreased histone acetylation and increased H3K9Me3 at the Fas promoter and increased their expression of Fas in the presence of an HDAC inhibitor. These findings demonstrate the critical role of histone modifications in the development of fibroblast resistance to apoptosis in both a murine model and in patients with pulmonary fibrosis and suggest novel approaches to therapy for progressive fibroproliferative disorders.

Normal wound healing is characterized by the recruitment and activation of fibroblasts to the areas of injury, followed by the clearance of fibroblasts after tissue has been repaired.^1^ The apoptosis of fibroblasts during this latter stage is felt to be critical in the prevention of pathological scarring.^2^ Undesired fibrosis can occur when activated fibroblasts persist in tissue and are not cleared by usual apoptotic mechanisms. Indeed, an overabundance of fibroblasts^3^ and fibroblast resistance to apoptosis^4, 5, 6, 7^ are hallmarks of many fibrotic disorders. Overcoming apoptosis resistance and inducing apoptosis in fibroblasts may serve as effective strategies for the treatment of many chronic fibroproliferative disease which, by some estimates, accounts for up to 45% of all deaths in the developed world.^8^

Idiopathic pulmonary fibrosis (IPF) is a fibrotic disorder of the lungs that exacts significant morbidity and mortality.^9, 10^ Lung fibroblasts and myofibroblasts are the major effector cells responsible for the excessive extracellular matrix production and scarring associated with IPF.^11^ Several studies have shown that fibroblasts from IPF patients are resistant to apoptosis,^12, 13^ and in particular Fas-mediated apoptosis,^4, 6, 7, 14, 15^ through a number of potential mechanisms, including decreased expression of the surface receptor Fas.^4, 16^ Activation of Fas (CD95, Apo-1, TNF receptor superfamily member 6) by either soluble or cell surface-expressed Fas ligand (FasL) results in the formation of the intracellular death complex domain and activation of caspase 8, which lead to apoptosis.^17^ The failure to activate Fas and induce fibroblast apoptosis has been shown to have a critical role in the pathogenesis of pulmonary fibrosis.^18, 19^ The mechanism for the resistance of IPF fibroblasts to Fas-mediated apoptosis and for the decreased expression of Fas is unknown.

Although epigenetic alterations, including DNA methylation and histone modifications, have been implicated in the dysregulation of genes associated with pulmonary fibrosis,^20, 21, 22, 23^ there are no studies that have examined whether epigenetic mechanisms are responsible for the apoptosis resistance of fibroblasts in IPF. Methylation of DNA is associated with decreased gene expression, whereas acetylation of histones results in relaxation of chromatin facilitating gene transcription. These and other histone modifications may function independently or in concert to regulate overall gene expression. Here, we investigated the mechanisms associated with the decreased expression of Fas in the lung fibroblasts of mice with bleomycin-induced pulmonary fibrosis and of patients with IPF and identified several histone modifications associated with the *FAS* (*TNFRSF6*) gene promoter that are responsible for the decreased expression of Fas and resistance to Fas-mediated apoptosis.

## Results

### Fibroblasts from the lungs of mice with bleomycin-induced fibrosis exhibit diminished expression of Fas and resistance to Fas-mediated apoptosis

**Figure 1:**
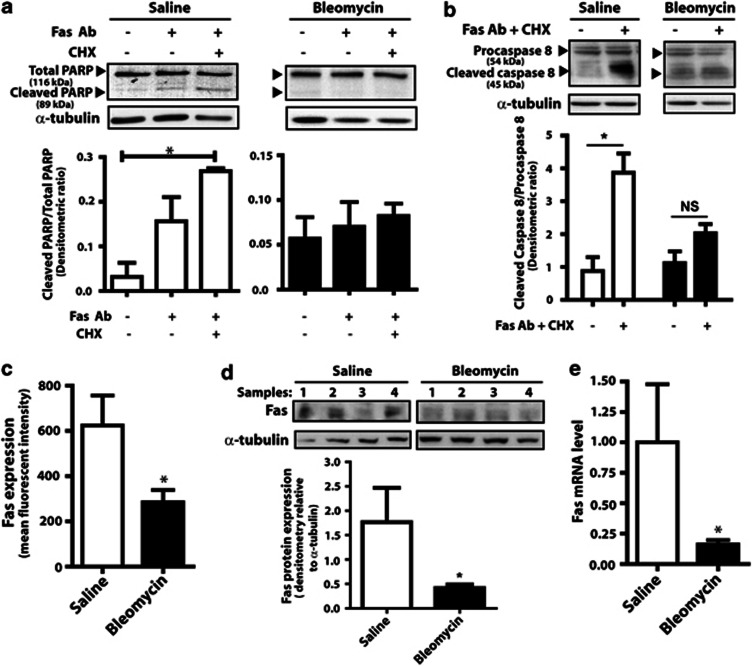
Fibroblasts from the fibrotic lungs of bleomycin-injured mice are resistant to Fas-mediated apoptosis and exhibit decreased expression of Fas. Fibroblasts from mice treated with bleomycin or saline control were treated with the anti-Fas activating antibody (Fas Ab, 100 ng/ml) and cycloheximide (CHX, 0.5 *μ*g/ml). Apoptosis was assayed by immunoblotting for (**a**) cleaved PARP and (**b**) cleaved caspase 8. Representative blot is shown, with densitometric ratio of cleaved to total PARP (*n*=3) and cleaved caspase 8 to procaspase 8 (*n*=4) shown below each blot. (**c**) Expression of cell-surface Fas in fibroblasts from bleomycin- and saline-treated mice was assayed by flow cytometry, with results expressed as mean fluorescent intensity (*n*=3 for saline, *n*=4 for bleomycin). (**d**) Total Fas protein expression (*n*=4) and (**e**) Fas mRNA levels (*n*=4 for saline, *n*=6 for bleomycin) were assayed as described in Methods. *P<0.05. NS, not significant

Administration of intratracheal bleomycin in C57Bl/6 mice induces pulmonary fibrosis, which is evident by days 14–21,^24^ and is a commonly used model of human fibrotic lung disease. Fibroblasts cultured from the fibrotic lungs of bleomycin-exposed mice have been shown to exhibit resistance to apoptosis,^4, 6, 7, 12, 13, 14^ but the mechanism(s) for apoptosis resistance is unclear. Fas-mediated apoptosis can be triggered *in vitro* by the addition of Fas-activating antibody and enhanced by the addition of cycloheximide, which prevents the synthesis of apoptosis inhibitor proteins.^14^ Fibroblasts cultured from the fibrotic lungs of mice 21 days after bleomycin exposure exhibited less apoptosis in response to Fas activation than did cells from saline-treated control mice (Figure 1). This resistance to Fas-mediated apoptosis was confirmed by assays of cleaved poly-ADP ribose polymerase (PARP) (Figure 1a) and cleaved caspase 8 (Figure 1b), both of which are diminished in fibroblasts from bleomycin-treated mice compared with saline-treated controls. To determine the mechanism for resistance to Fas-mediated apoptosis, we examined the expression of Fas and observed that cell surface expression of Fas (as assayed by flow cytometry), total protein expression of Fas from whole cell lysates (as determined by immunoblot), and Fas mRNA levels were all decreased in fibroblasts from bleomycin-treated mice compared with saline-treated controls (Figures 1c–e). These findings suggest that diminished expression of Fas may be responsible for the resistance to Fas-mediated apoptosis.

### Diminished expression of Fas in fibroblasts from bleomycin-injured mice is attributed to histone modifications

DNA methylation and histone modifications have been shown to be responsible for the differential expression of certain genes in pulmonary fibrosis.^20, 22, 23^ To examine whether such epigenetic mechanisms are responsible for the diminished expression of Fas in fibroblasts from bleomycin-injured mice, we first examined levels of DNA methylation near the transcription start site of Fas. Performing bisulfite sequencing, we observed no difference in DNA methylation between fibroblasts from fibrotic and nonfibrotic lung at any of the 16 CpG loci analyzed (Supplementary Figure S1).

**Figure 2:**
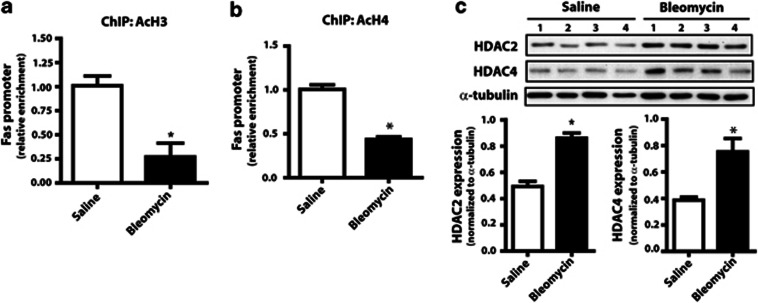
Fibroblasts from bleomycin-injured mice are associated with decreased histone acetylation. ChIP using antibodies against (**a**) acetylated H3 (*n*=6 for saline, *n*=5 for bleomycin) and (**b**) acetylated H4 (*n*=6 for saline, *n*=4 for bleomycin) was performed in fibroblasts from saline- and bleomycin-treated mice. Relative enrichment of the Fas promoter, normalized to saline control, was assayed by real-time PCR from immunoprecipitated DNA. (**c**) Expression of HDAC2 and HDAC4 were assayed by immunoblot in fibroblasts from bleomycin- and saline-treated mice, with mean densitometry values shown (*n*=4). *P<0.05

We next examined whether fibroblasts from bleomycin-treated mice exhibit differences in histone modifications associated with the *Fas* promoter. We performed chromatin immunoprecipitation (ChIP) using antibodies to acetylated H3 and acetylated H4 and observed that the *Fas* promoter in fibroblasts from bleomycin-injured mice manifested decreased H3 (Figure 2a) and H4 (Figure 2b) acetylation. More than 11 isoforms of the classical histone deacetylase (HDAC) family, separated phylogenetically into two classes, are capable of deacetylating histones with varying specificity.^25^ Fibroblasts from the lungs of fibrotic mice were observed to exhibit increased levels of both HDAC2, a class I HDAC, and HDAC4, a class II HDAC (Figure 2c).

**Figure 3:**
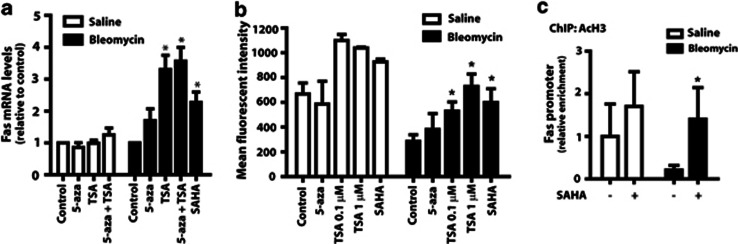
Treatment of fibroblasts from bleomycin-injured mice with HDAC inhibitors resulted in increased expression of Fas. Fibroblasts from saline- and bleomycin-treated mice were treated for 72 h with the DNA methylation inhibitor 5-aza-dC (1 *μ*M) or the HDAC inhibitors TSA (0.1 *μ*M unless otherwise specified) and SAHA (1 *μ*M) and (**a**) assayed for Fas mRNA levels by real-time RT-PCR (*n*=4 for saline, *n*=6 for bleomycin; normalized to untreated control) and (**b**) cell surface expression by flow cytometry (*n*=2 for saline, *n*=4 for bleomycin). (**c**) ChIP for acetylated H3 was performed in cells pretreated with or without SAHA (*n*=3 for saline, *n*=4 for bleomycin). Enrichment for the Fas promoter was assayed by PCR and normalized to untreated saline. *P<0.05 relative to untreated control

To determine whether the deacetylation of histones and the increase in HDACs in fibroblasts from bleomycin-injured mice are sufficient to cause diminished expression of Fas, we treated cells with two nonselective HDAC inhibitors, trichostatin A (TSA) and suberoylanilide hydroxamic acid (SAHA). Treatment with either of the HDAC inhibitors, but not the DNA methylation inhibitor 5-aza-2′-deoxycytidine (5-aza-dC), resulted in a significant increase in Fas mRNA levels in cells from mice with bleomycin-induced fibrosis (Figure 3a). Neither TSA nor 5-aza-dC had any effect on Fas mRNA levels in fibroblasts from saline-control mice. Treatment of fibroblasts from bleomycin-treated mice with TSA or SAHA, but not 5-aza-dC, increased cell surface expression of Fas, as assayed by flow cytometry, to levels comparable with that observed in fibroblasts from saline-treated mice (Figure 3b). Total Fas protein expression in lung fibroblasts from bleomycin-treated mice was likewise increased in the presence of TSA or SAHA (data not shown). Treatment with SAHA furthermore resulted in a selective increase of acetylated histones at the Fas promoter in cells from bleomycin-injured mice but not in those from saline controls (Figure 3c).

**Figure 4:**
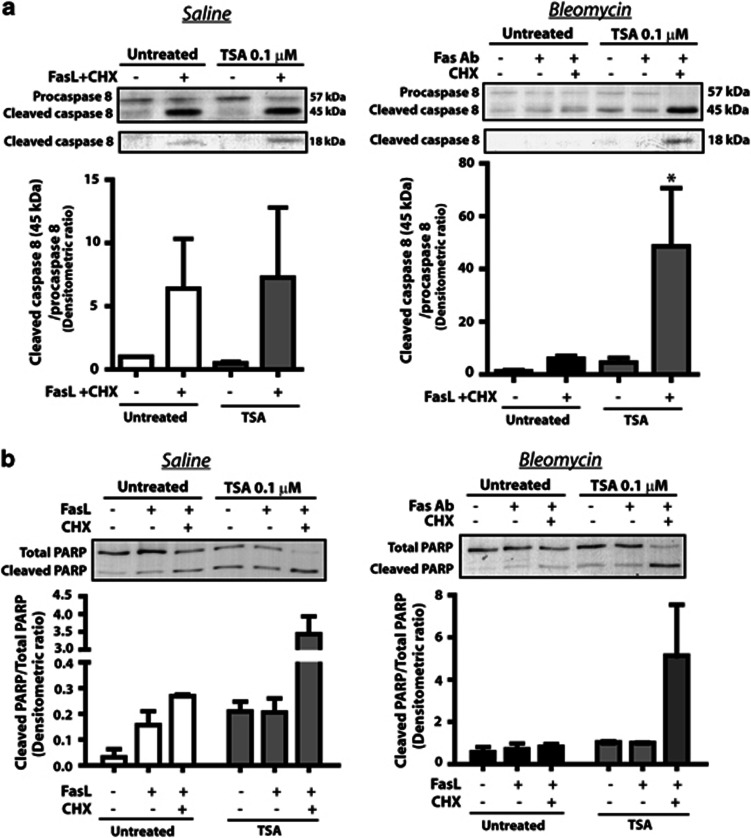
Pretreatment of fibroblasts from bleomycin-injured mice with TSA increased cell susceptibility to Fas-mediated apoptosis. Fibroblasts from the lungs of saline- and bleomycin-treated mice were pretreated with or without TSA (0.1 *μ*M) for 72 h before being treated with the anti-Fas activating antibody (100 ng/ml) and cycloheximide (CHX, 0.5 *μ*g/ml). (**a**) Cleaved caspase 8 and (**b**) cleaved PARP were assayed by immunoblot, with mean densitometric ratio of cleaved caspase 8 to procaspase 8 (*n*=4 for both saline and bleomycin) and cleaved PARP to total PARP (*n*=2 for saline, *n*=3 for bleomycin) shown below each representative blot. *P<0.05

We next pretreated fibroblasts from saline-control and bleomycin-injured mice with TSA and examined the susceptibility of the cells to apoptosis by the Fas-activating antibody and cycloheximide. Fibroblasts from bleomycin-injured mice pretreated with TSA exhibited increased susceptibility to apoptosis by Fas-activating antibody and cycloheximide, as assayed by levels of cleaved caspase 8 (Figure 4a) and cleaved PARP (Figure 4b). TSA pretreatment of fibroblasts from saline-control mice did not alter the ability of Fas-activating antibody and cycloheximide to induce apoptosis in these already apoptosis-sensitive cells. Together, these results suggest that histone deacetylation is responsible for the decreased Fas expression and apoptosis resistance observed in fibrotic fibroblasts.

**Figure 5:**
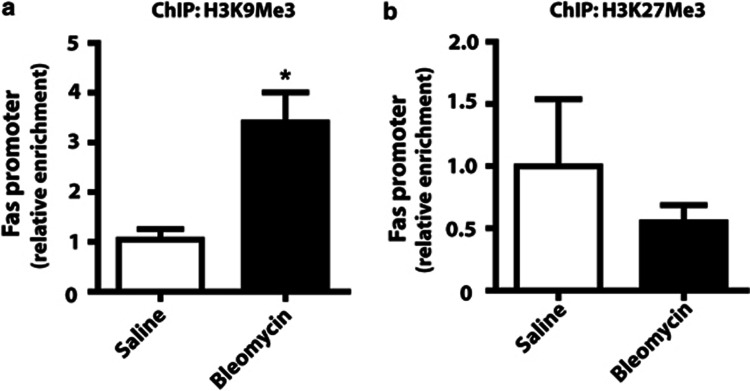
Fibroblasts from bleomycin-treated mice were associated with increased H3K9Me3 at the Fas gene promoter. ChIP using antibodies against (**a**) H3K9Me3 (*n*=3) and (**b**) H327Me3 (*n*=6) was performed in fibroblasts from saline- and bleomycin-treated mice. Enrichment for the Fas promoter normalized to saline control was assayed by real-time PCR. *P<0.05

Histone acetylation/deacetylation is often associated with histone methylation changes at specific histone residues.^26^ These latter modifications may be either the cause or effect of histone deacetylation. In particular, histone 3 lysine 9 trimethylation (H3K9Me3) and histone 3 lysine 27 trimethylation (H3K27Me3) are associated with decreased gene expression and the recruitment of HDACs.^26^ ChIP analysis using antibodies to H3K9Me3 and H3K27Me3 revealed that the Fas promoter is associated with increased H3K9Me3, but not H3K27Me3, in fibroblasts from bleomycin-injured mice compared with saline-control mice (Figure 5).

### Histone modifications are also responsible for the decreased expression of Fas in fibroblasts from IPF patients

**Figure 6:**
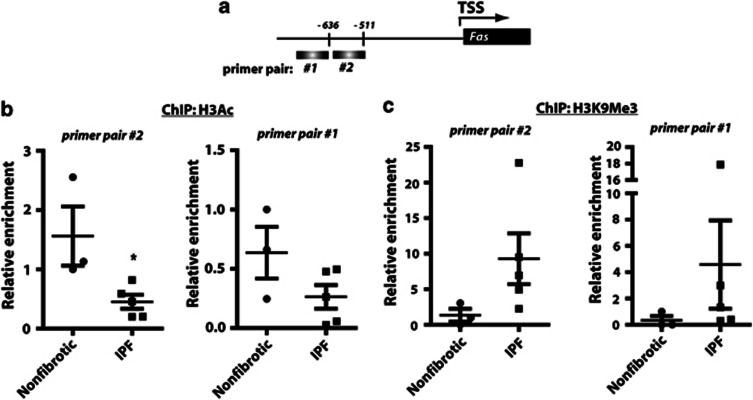
The Fas gene promoter in IPF fibroblasts was also associated with decreased histone acetylation and increased H3K9 trimethylation. ChIP using antibodies against (**b**) acetylated H3 and (**c**) H3K9Me3 was performed in fibroblasts from IPF patients and nonfibrotic controls. (**a**) Immunoprecipitated DNA was PCR amplified using primer pairs from two separate regions of the Fas gene promoter. *P<0.05

**Figure 7:**
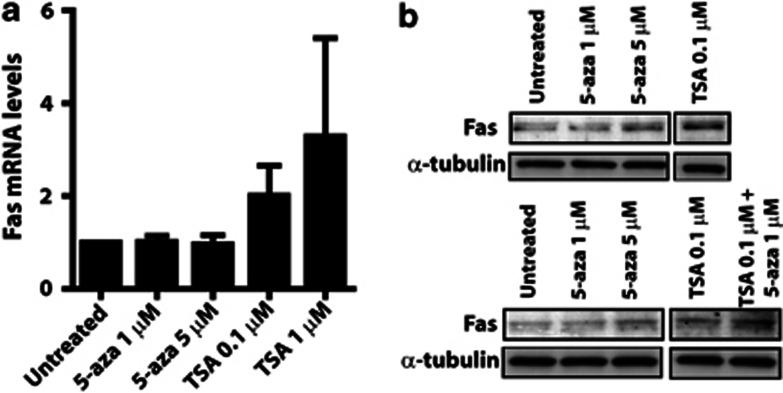
Treatment of IPF fibroblasts with TSA increased Fas expression. IPF fibroblasts were treated with the DNA methylation inhibitor 5-aza-dC or the HDAC inhibitor TSA at the respective concentrations and (**a**) assayed for Fas mRNA levels (*n*=3) and (**b**) protein expression by immunoblot

Fas expression has been shown to be decreased in lung fibroblasts from patients with IPF, and diminished Fas expression in IPF fibroblasts is associated with increased resistance to Fas-mediated apoptosis^4, 16^. The mechanisms responsible for decreased Fas expression in IPF fibroblasts, however, are unknown. Based on our observations in the bleomycin mouse model of fibrosis, we sought to determine whether histone modifications are also responsible for the decreased expression of Fas in cells from IPF patients. We performed ChIP utilizing antibodies to acetylated H3 and H3K9Me3 and assayed by real-time PCR two regions within the FAS gene promoter. Fibroblasts from IPF patients exhibited decreased histone acetylation and increased H3K9Me3 at both regions interrogated along the gene promoter (Figure 6). Treatment of IPF fibroblasts with the HDAC inhibitor TSA, but not the DNA methylation inhibitor 5-aza-dC, resulted in increased Fas mRNA (Figure 7a) and protein expression (Figure 7b). There was no difference in Fas promoter DNA methylation between IPF and nonfibrotic control fibroblasts (Supplementary Figure S1b). These data suggest that histone deacetylation and trimethylation of lysine 9 on H3 are responsible for the decreased Fas expression in IPF fibroblasts as also observed in bleomycin fibroblasts.

## Discussion

Although the recruitment, expansion, and activation of fibroblasts at areas of tissue injury are considered integral to normal wound healing, apoptosis of fibroblasts is also critical for the eventual clearance of these cells after wound healing resolves.^1, 2^ The persistence of activated fibroblasts beyond this period is felt to contribute to pathologic scarring, and fibroblast resistance to apoptosis is a key hallmark of many fibrotic disorders. The mechanism(s) for this resistance is, in most cases, unknown. Here, utilizing an animal model of pulmonary fibrosis and examining primary cells from patients with IPF, we identified histone modifications – specifically histone deacetylation and H3K9Me3 – that are responsible for the decreased expression of the death receptor Fas and resistance to Fas-mediated apoptosis in fibroblasts from fibrotic lung. In both the mouse model and the human disease, treatment with HDAC inhibitors increased Fas expression and restored sensitivity of fibrotic cells to Fas-mediated apoptosis.

Changes in DNA methylation and histone modifications have been shown to modulate the expression of several genes in pulmonary fibrosis;^20, 21, 22, 23^ however, this is the first demonstration of histone modifications being responsible for the specific downregulation of Fas and the resistance of fibrotic lung fibroblasts to Fas-mediated apoptosis. Although the function of Fas was originally described in studies of adaptive immunity,^27, 28^ the importance of Fas–FasL interactions in other diseases, including pulmonary fibrosis, are well substantiated.^18, 19^ It was originally suggested from indirect studies employing HDAC inhibitors in cancer that Fas expression may be regulated by histone acetylation.^29, 30^ More recent studies have shown that diminished Fas expression is associated with histone deacetylation in models of colon and liver inflammation.^31, 32^ IPF is a disease of unknown etiology but is thought to be influenced by certain environmental exposures, some of which may affect epigenetic machinery.^33^ The persistence of apoptosis resistance in fibrotic lung fibroblasts through serial passages in culture further suggests that epigenetic mechanisms are responsible for these changes. Our studies presented here highlight the importance of histone modifications in the resistance of fibrotic lung cells to apoptosis and in their role in the pathogenesis of pulmonary fibrosis.

Although we identified histone modifications to be the mechanism for the diminished expression of Fas in pulmonary fibrosis, how these modifications arise at the Fas promoter is unknown. This process is presumably acquired, as these modifications were observed in murine models of pulmonary fibrosis in which fibrosis develops after bleomycin injury. The histone modifications are furthermore heritable with cell division, as they are sustained in cell culture over several passages. It has been shown that Fas overexpression and increased expression of Fas by the cytokines tumor necrosis factor-*α* and interferon-*γ* are sufficient to overcome the resistance of IPF fibroblasts to apoptosis.^16, 34^ Further studies would be needed to determine whether this cytokine-mediated increase in Fas expression is a result of modifications to histones. The study by Wynes *et al.*^16^ did show that the cytokine-mediated increase in Fas depends on the transcription factor NF-κB, which recruits histone acetyltransferases to sites of transcription.^35^

In addition to histone deacetylation, we also observed that the Fas gene promoter in fibrotic cells exhibited an increase in H3K9Me3, but not H3K27Me3. Both H3K9Me3 and H3K27Me3 marks are associated with gene silencing and have been implicated as drivers of other chromatin changes, including histone deacetylation and DNA methylation.^26^ Interestingly, we did not observe any difference in DNA methylation at the Fas gene promoter in fibrotic lung fibroblasts from mice or IPF patients, and it is unclear in this case what the exact relationship is between H3K9Me3 and histone deacetylation in regulating Fas expression in fibrotic lung fibroblasts. Others investigators have reported that H3K9Me3 marks may be a consequence, rather than a determinant, of histone deacetylation,^36^ and in our case, treatment of fibroblasts with HDAC inhibitors was sufficient to restore Fas expression and cell susceptibility to apoptosis. Fibrotic lung fibroblasts were also observed to exhibit increased expression of HDAC2 and HDAC4, suggesting that increased expression of HDACs may be sufficient for the deacetylation of histones and decreased expression of Fas. The presence of H3K9Me3, however, may reinforce gene silencing and promote the heritability of chromatin changes during cell division.

Apoptosis requires the complex interplay of many signaling molecules, and the deficiency or overexpression of other apoptosis regulatory proteins besides Fas has also been implicated in the resistance of fibroblasts to apoptosis in the setting of pulmonary fibrosis.^14, 15^ In fact, our finding that Fas expression is diminished in fibroblasts from bleomycin-injured mice differs from that of Wallach-Dayan *et al.*,^7^ who did not observe a difference in Fas expression between fibroblasts from bleomycin-injured mice and controls. This discrepancy may be attributed to the fact that Wallach-Dayan *et al.*^7^ studied fibroblasts that were cultured from mice just 7 days after bleomycin-induced pulmonary injury. Because pulmonary fibrosis typically develops at days 14–21 post-bleomycin exposure, the fibroblasts that we used in our experiments (taken from mice at day 21 post-bleomycin treatment) may be phenotypically different from those used in the study by Wallach-Dayan *et al.*^7^ and may illustrate the plasticity of histone modifications in fibroblasts during the evolution of fibrosis. These as well as other investigators identified increased expression of cellular FLICE-like inhibitory protein (c-FLIP) as also contributing to the resistance of fibroblasts to apoptosis,^15, 37^ although this was not consistently observed among fibroblasts in IPF tissue.^38^ Increased expression of inhibitors of apoptosis such as survivin^14, 39^ and X-linked inhibitor of apoptosis (XIAP)^15^ may also have a substantial role in apoptosis resistance. Indeed, the ability of cycloheximide to augment Fas-mediated apoptosis is thought to reflect the importance of endogenous production of inhibitors of apoptosis. It would be informative to determine whether histone modifications are also responsible for altered expression of c-FLIP, survivin, or XIAP in pulmonary fibrosis.

In our studies, the decreased Fas expression in fibrotic lung fibroblasts was observed at the transcriptional level that is congruent with its expression being regulated by epigenetic mechanisms. However, cell surface expression of Fas has also been reported to be regulated by post-translational mechanisms,^4^ and these mechanisms can be regulated by oxidative stress.^40^ Furthermore, fibroblast expression of FasL has been shown to promote epithelial cell apoptosis, which is also considered critical to the pathogenesis of IPF.^41, 42^ Whether FasL expression is regulated by histone modifications in pulmonary fibrosis is unknown.

Although bleomycin administration is a commonly used mouse model of pulmonary fibrosis, there are substantial limitations in its ability to approximate IPF. Nonetheless, in terms of Fas-mediated resistance to apoptosis, we observed similar mechanisms of histone modifications in the bleomycin model as in the human disease. We found that the degree of histone deacetylation and H3K9 trimethylation as well as responsiveness to HDAC inhibitors varied among individual IPF patient lines. This is not surprising given the phenotypic heterogeneity of IPF fibroblasts observed in other contexts.^39, 43^

IPF is a devastating lung disease with no clearly effective therapy aside from lung transplantation.^10^ Fibroblasts are the major effector cells responsible for the generation of extracellular matrix proteins that comprise scar tissue and are abundant within fibroblastic foci, the characteristic pathological lesion in IPF. Stimulation of fibroblast apoptosis is thus an appealing therapeutic approach, but fibroblast resistance to apoptosis poses a potential barrier to such therapy. The identification of histone modifications as responsible for this resistance and the ability of HDAC inhibitors to overcome this resistance represent novel avenues for future therapy of this deadly disease.

## Materials and Methods

### Cells

Bleomycin (0.0010 U/kg body weight) was administered intratracheally to C57Bl/6 mice (Charles River Laboratory, Wilmington, MA, USA) to induce pulmonary fibrosis, which develops by days 14–21.^24^ Administration of intratracheal saline was used as control. At day 21, mice were euthanized and the lungs were minced and placed in Dulbecco's Modified Eagle Medium (DMEM, Invitrogen, Carlsbad, CA, USA) supplemented with 10% fetal bovine serum (Hyclone, Logan, UT, USA) and penicillin/streptomycin (Invitrogen). Outgrowths of fibroblasts from minced lungs were cultured, washed, and passaged for use in subsequent experiments.

Fibroblasts were also cultured from the outgrowths of lung tissue obtained by surgical lung biopsy of patients with IPF as previously described.^43^ All segments were confirmed histologically to show usual interstitial pneumonia. Nonfibrotic control fibroblasts were cultured from histologically normal regions of the lung from patients undergoing resection for lung nodules. Fibroblasts were cultured in DMEM supplemented with 10% FBS and antibiotics.

Cells were treated with the DNA methylation inhibitor 5-aza-dC (1–5 *μ*M, Sigma-Aldrich, St Louis, MO, USA) or the HDAC inhibitors TSA (0.1 *μ*M–1 *μ*M, Sigma-Aldrich) and SAHA (1 *μ*M, Cayman Chemical, Ann Arbor, MI, USA) for 72 h before being harvested for expression of Fas. For apoptosis experiments, cells were pretreated with or without TSA 0.1 *μ*M for 48 h, before the medium was removed and cells were treated with the anti-Fas-activating antibody (100 ng/ml; clone CH11 Millipore, Billerica, MA, USA for human cells and clone Jo2 No.554248 BD Biosciences, Sparks, MD, USA for mouse cells) and cycloheximide (0.5 *μ*g/ml, Sigma-Aldrich).

### Bisulfite sequencing

The DNA methylation of the Fas promoter was assayed by bisulfite sequencing. DNA was isolated from cells using the DNeasy kit (Qiagen, Valencia, CA, USA) and subjected to bisulfite conversion using the Zymo EZ DNA Methylation kit (Zymo Research, Irvine, CA, USA) as per the manufacturer's protocol. Regions of the Fas promoter were PCR amplified using biotin-labeled primers obtained from EpigenDx, Inc (Worcester, MA, USA) specific for human and murine sequences. The PCR product was isolated by sepharose beads, washed, and sequenced using specific sequencing primers (EpigenDx, Inc) on the Q24 pyrosequencer (Qiagen).

### ChIP

A total of 9 × 10^6^ cells were cultured on three 100 mm plates. Cells were incubated for 5 min in 1% formaldehyde to establish DNA-chromatin crosslinks, washed, and subsequently incubated in glycine to stop further fixation. Nuclear material was isolated using nonionic detergent buffers, and chromatin was sheared to 200–500 bp fragments by 15 min of sonication using the Covaris sonicator (Covaris, Inc, Woburn, MA, USA), as per the manufacturer's protocol. Chromatin was incubated overnight with the following antibodies: anti-acetylated H3 (25 *μ*g/ml, Nos.17–615 Millipore), anti-acetylated H4 (25 *μ*g/ml, Nos.06–598 Millipore), anti-H3K9Me3 (20 *μ*g/ml, Nos.17–658 Millipore), and anti-H3K27Me3 (1 : 50, Nos.9733 Cell Signaling, Beverly, MA, USA). ChIP was performed using magnetic beads from the ActiveMotif ChIP-IT Express Kit (ActiveMotif, Carlsbad, CA, USA) according to the manufacturer's protocol. Immunoprecipitated chromatin was reverse cross-linked and DNA was cleaned using the DNA cleanup kit (Qiagen). Isolated DNA was PCR amplified by real time using primers predesigned to amplify a region 1 kilobases upstream of the transcription start site of the mouse Fas promoter (No.334001 GPM1034075(−)01A, Qiagen). Primers for the human Fas promoter include pair No.1: 5′-ATAGCTGGGGCTATGCGATT-3′ (forward primer), 5′-GTTGTGGCTGCAACATGAGA-3′ (reverse primer); and human Fas promoter primer pair No.2: 5′-GTGAGCCTCTCATGTTGCAG-3′ (forward primer), 5′-GTTGGGGAGGTCTTGAAGGA-3′ (reverse primer).

### Real-time RT-PCR

Levels of Fas mRNA were assayed by real time RT-PCR. RNA was isolated from cells using Trizol (Invitrogen) and quantitative mRNA levels were assayed by real-time RT-PCR using the Applied Biosystems 7300 Real-time PCR System (Carlsbad, CA, USA). Primers for human and murine Fas were obtained from Applied Biosystems. Primer and probes for human and murine β-actin were used as previously reported.^44^

### Immunoblotting

Cell lysates were collected in lysis buffer (PBS containing 1% Nonidet P-40, 0.5% sodium deoxycholate, 0.1% SDS, 2 mM orthovanadate, and protease cocktail inhibitor), resolved by SDS-PAGE, transferred to nitrocellulose membranes, and immunoblotted using antibodies to the following: PARP (1 : 1000, No.9542 Cell Signaling), caspase 8 (1 : 1000, No.9429 Cell Signaling), murine Fas (1 : 1000, No.554258 BD Biosciences), human Fas (1 : 500, No.05–201 Millipore), HDAC2 (1 : 2000, No.17–10237 Millipore), HDAC4 (1 : 2000, No.7628 Cell Signaling) and *α*-tubulin (1 : 1000, Sigma-Aldrich). Bound primary antibodies were visualized with appropriate secondary antibody conjugated to horseradish peroxidase and developed with enhanced chemiluminescence reagent (GE Healthcare, Piscataway, NJ, USA). Densitometric analysis was performed on the visualized bands using Scion Image Software (NIH, Bethesda, MD, USA).

### Flow cytometry

Cultured cells were scraped from plates, washed in PBS, and incubated with PE-labeled antibody to Fas (clone Jo2 No.554248 BD Biosciences) for 15 min at 4 ° C. Cells were subsequently washed three times and analyzed for fluorescence by flow cytometry on the FACSCalibur (BD Biosciences).

### Statistical analysis

Data were analyzed on GraphPad Prism 5.0 (GraphPad Prism Software, San Diego, CA, USA) using ANOVA or Student's *t*-test, as appropriate, with a *P*<0.05 defined as statistically significant. Data are expressed as mean±S.E.M.

## Abbreviations

IPF: idiopathic pulmonary fibrosis
FasL: Fas ligand
PARP: poly-ADP ribose polymerase
ChIP: chromatin immunoprecipitation
HDAC: histone deacetylase
5-aza-dC: 5-aza-2′-deoxycytidine
TSA: trichostatin A
SAHA: suberoylanilide hydroxamic acid
H3K9Me3: histone 3 lysine 9 trimethylation
H3K27Me3: histone 3 lysine 27 trimethylation
c-FLIP: cellular FLICE-like inhibitory protein
XIAP: X-linked inhibitor of apoptosis
