# Supplementary figures and images for: Histone modifications are responsible for decreased Fas expression and apoptosis resistance in fibrotic lung fibroblasts
Source: Cell Death Dis. 2013 May 2;4(5):e621–. doi: 10.1038/cddis.2013.146 (PMC3674355; doi:10.1038/cddis.2013.146)

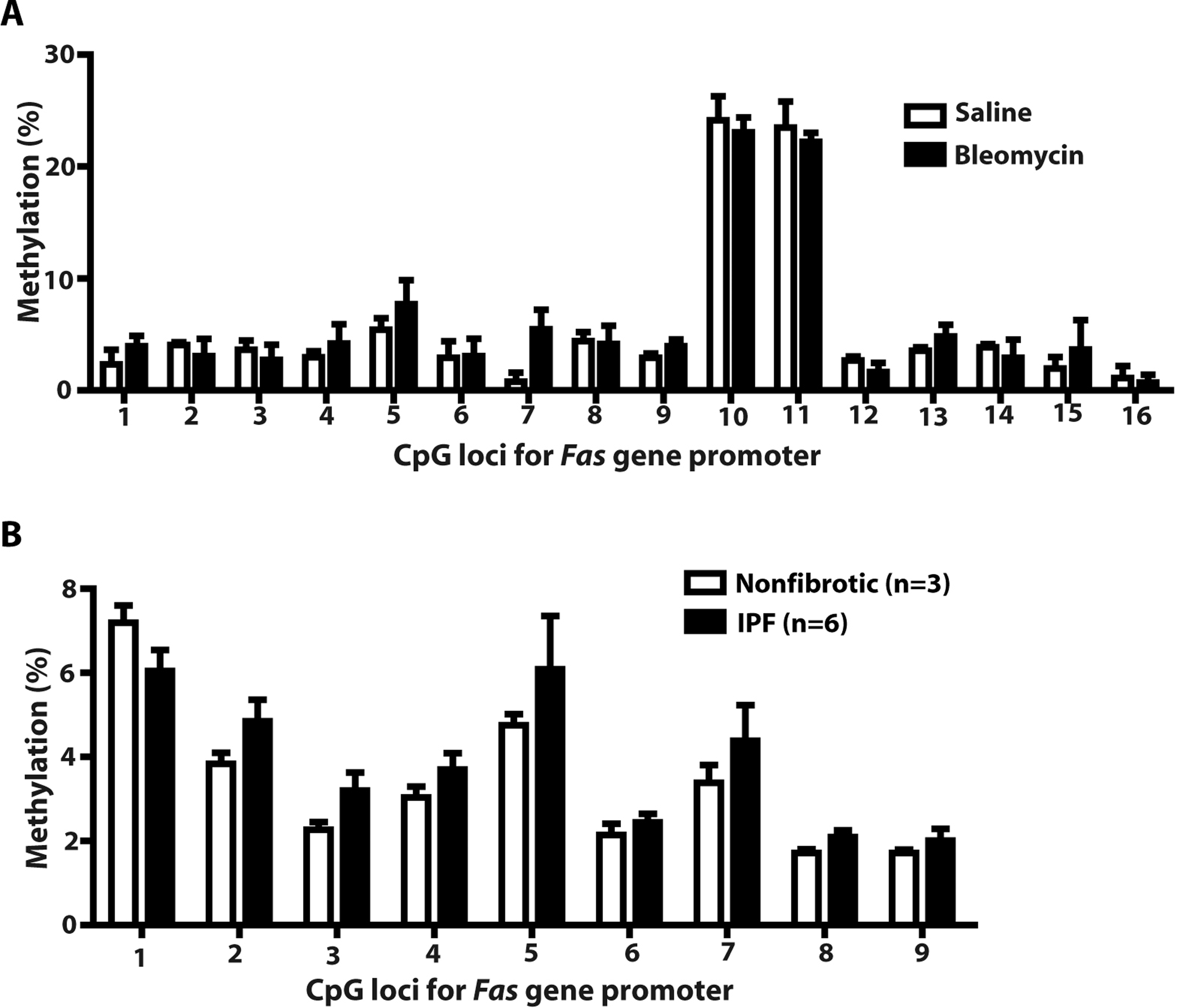

Supplement: Supplementary Figure S1 [file cddis2013146x1.tif]
