# Supplementary material for: Histone modifications are responsible for decreased Fas expression and apoptosis resistance in fibrotic lung fibroblasts
Source: Cell Death Dis. 2013 May 2;4(5):e621–. doi: 10.1038/cddis.2013.146 (PMC3674355; doi:10.1038/cddis.2013.146)
Supplement: Supplementary Figure Legend [file cddis2013146x2.doc]

**Supplemental Figure 1** DNA methylation of the Fas gene promoter in fibrotic versus nonfibrotic lung fibroblasts. (a) DNA methylation of 16 CpG loci within the murine Fas gene promoter was assayed in fibroblasts from bleomycin- and saline-treated mice (n=3). (b) DNA methylation of 9 CpG loci within the human Fas gene promoter was assayed in IPF and nonfibrotic lung fibroblasts.
